# Mistaking a Tumour for an Infection - Acrometastasis of the Finger from Endocervical Adenosquamous Carcinoma: A Case Report

**DOI:** 10.5704/MOJ.2111.017

**Published:** 2021-11

**Authors:** AS Yong, PS Gill, A Shalimar, J Sapuan

**Affiliations:** Department of Orthopaedics and Traumatology, Universiti Kebangsaan Malaysia, Kuala Lumpur, Malaysia

**Keywords:** acrometastasis, metastasis, hand infection, endocervical adenosquamous carcinoma, terminal presentation

## Abstract

Acrometastasis is rare with a very low incidence of all bone metastasis. It can present with swelling, pain and warmth with erythema that may mimic an infection especially in the distal phalanx. Due to its rarity and subtle clinical presentation, it can be misdiagnosed as an infection causing the treatment to be delayed. We report a 42-year-old female with an acrometastasis to the distal phalanx of the left middle finger which we mistook as an infection thus delaying her treatment. It was a terminal presentation of her endocervical adenosquamous carcinoma. We would like to highlight that acrometastasis has an indistinct presentation and in cases where the lesion does not respond to treatment, acrometastasis should be included as one of the differential diagnoses. Thus, physicians need to have a high level of suspicion in patients with a primary malignant tumour.

## Introduction

Acrometastasis is defined as metastasis distal to the elbow and knee. It has a rare occurrence with only 0.1% of cases reported^1-4^. The most common sites for the primary tumours are namely – lung, colorectal, breast and genitourinary. Acrometastasis from endocervical adenosquamous carcinoma is extremely uncommon^4^. Though rare, they are an indicator of poor prognosis, and survival has been reported to be only up to seven months from diagnosis^1,3,4^. They can present as a subtle swelling, with or without pain and sometimes they present as an infection to the fingers following trivial trauma. A high index of suspicion should be present when the patient has a known history of malignancy. Despite that, acrometastasis could be a terminal presentation of an undiagnosed primary malignancy^1-4^. An accurate history, clinical examination and adequate imaging are needed to diagnose acrometastasis.

We would like to present a case of acrometastasis to the finger that has been misdiagnosed as an infection.

## Case Report

**Fig. 1:**
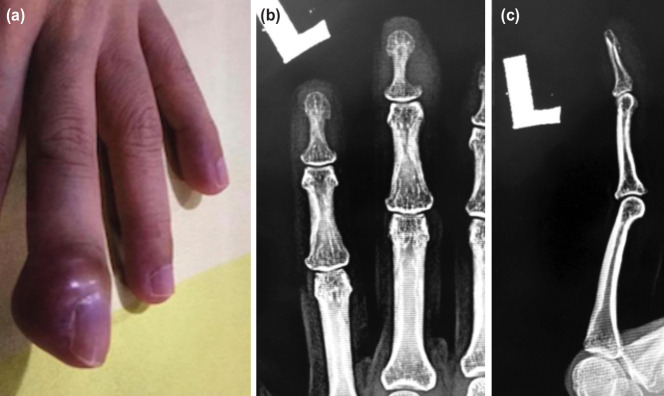
(a) Clinical picture of the left middle finger swelling and erythema mimicking an infection, (b and c) Lateral and AP radiograph of the left middle finger showing increase in soft tissue shadow and scalloping of the distal phalanx at two and half months after the onset of symptoms.

A 42-year-old female with a known case of endocervical adenosquamous carcinoma, presented with a complaint of progressive swelling and pain over her left middle finger for two months. She had been diagnosed with endocervical adenosquamous carcinoma stage 3 six months ago and had undergone 27 cycles of radiotherapy and chemotherapy. She has no metastasis to the bone or other organs. It was treated as paronychia – an incision and drainage procedure was performed and she was discharged with antibiotics. Two weeks later she returned with the swelling increasing in size. Radiographs showed scalloping of the distal phalanx with increase in soft tissue shadow of the middle finger (Fig. 1).

**Fig. 2:**
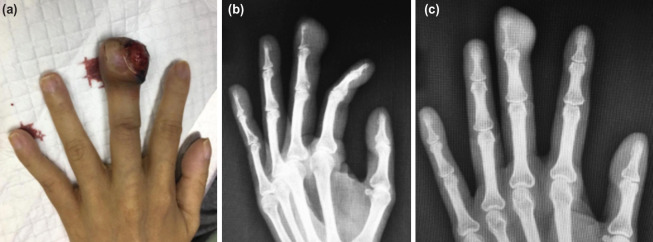
(a) Clinical picture of the left middle finger at three and a half months after onset of symptoms showing fungating mass at the radio-dorsal aspect of the middle finger extending to the distal crease, (b and c) AP and oblique radiograph of the left middle finger showing an eccentric lytic lesion not extending to the distal interphalangeal joint with increase in soft tissue shadow at the distal phalanx.

Another incision and drainage was performed and she was started on six weeks antibiotics course. However, she presented again three weeks later with increased swelling of the lesion with severe pain. Amputation was suggested however she sought a second opinion at our centre. Upon presentation, it was a fungating mass that has extended proximal to the distal finger crease (Fig. 2).

She also complained of severe upper lumbar pain which was investigated concurrently. MRI showed multiple metastases to the spine as well as to the bilateral lungs.

Subsequently, an MRI of the lesion was performed, and it showed a lobulated soft tissue mass extending from the distal phalanx and middle phalanx of the middle finger likely representing a locally aggressive tumour. HPE showed a metastatic lesion with primary from the endocervical adenosquamous carcinoma. She was planned for amputation of the middle finger; however, she developed superior vena cava obstruction and surgery was delayed due to her unstable conditions. In the end, she succumbed to her advanced-stage disease.

## Discussion

Acrometastasis can present as pain and swelling of the finger. Sometimes it can mimic an infection where the finger is swollen with erythema and pain and can further worsen with ulceration^1-2^. Any history of malignancies should be highlighted, and a high suspicion index should be maintained throughout. Radiographs usually show a lytic, sclerotic or mixed lesion depending on the primary malignancy. MRI is more useful where the marrow and soft tissue extension of the tumour can be evaluated^1^. Our patient presented with swelling and pain in the finger where an incision and drainage was done and she was started on antibiotics, however it did not resolve. With her history of previous malignancy, this should have triggered the attending physician of a possibility of acrometastasis. Unfortunately for this patient, her acrometastasis was a terminal sign of the primary tumour whereby the correct diagnosis was delayed. Although we knew that amputation was the solution, however we were debating on performing a proximal interphalangeal joint level amputation (for an infection) versus a Ray amputation (for a malignant tumour) causing another delay. Other differential diagnoses that need to be excluded are felon, gout, osteomyelitis, rheumatoid arthritis, pyogenic granuloma, tuberculosis and primary skin tumours^1,4^.

Acrometastasis is rare because bone metastasis happens at the area of rich red marrow in which the phalanx is void of^1,3,4^. Metastasis happens via hematogenous spread where malignant cells from lungs have easy access thru the arterial circulation of the upper limbs^1,3,4^. That explains why patients with primary lung malignancy or patients with lung metastasis from other malignancies have a higher incidence of acrometatasis compared to those without^1,3,4^. In our patient, multiple lung metastases might have been the source of the malignant cells rather than the primary lesion. The most common locations are the distal phalanx of the middle finger followed by the thumb.

Acrometastasis from the endometrium is very rare. From our search, there have only been two cases reported in the English literature^1^. Sur *et al*^2^ reported a case of acrometastasis from the uterus which, however, was a uterine leiomyosarcoma and Ornetti *et al*^5^ presented another case which was not reported formally.

Staging is needed when acrometastasis is suspected to determine primary lesion, extension and prognosis^1^. A tissue diagnosis via a biopsy is compulsory before options of treatment can be given^1^. Depending on the staging, options of treatments are amputation or wide local excision with reconstruction and subsequently radiotherapy and/or chemotherapy. We have planned an amputation for our patient with the aim of pain relief and preservation of hand function at the same time allows a wide margin resection^1^.

Prognosis is usually poor; the mean survival rate is less than six months^1-4^. There have been cases reported of survival rate of up to two years in renal carcinoma however this is rare^1^. Given the poor prognosis, acrometastasis is considered a terminal presentation of the primary malignancy^4^.

In conclusion, acrometastasis presentation can mimic an infection of the finger and in some circumstances, this is a terminal presentation of patients with primary malignant tumour. Therefore, a high index of suspicion must be given to patients with a history of malignancy. Accurate history and clinical examination lead to accurate diagnosis and can accelerate treatment. Despite the poor prognosis, amputation is the most common treatment to alleviate pain, return of function and improve patient's quality of life.
